# Onnamides A and B Suppress Hepatitis B Virus Transcription by Inhibiting Viral Promoter Activity

**DOI:** 10.3390/md24010021

**Published:** 2026-01-01

**Authors:** Yasuhiro Hayashi, Sei Arizono, Nanami Higa, Trianda Ayuning Tyas, Yuichi Akahori, Kenji Maeda, Masaaki Toyama, Kanami Mori-Yasumoto, Mina Yasumoto-Hirose, Kei Miyakawa, Junichi Tanaka, Takahiro Jomori

**Affiliations:** 1 Faculty of Agriculture, University of Miyazaki, 1-1 Gakuen-kibanadai-nishi, Miyazaki City 889-2192, Miyazaki, Japan; 2 Faculty of Science, University of the Ryukyus, 1 Senbaru, Nishihara City 903-0213, Okinawa, Japan; k238364@eve.u-ryukyu.ac.jp (N.H.); jtanaka@cs.u-ryukyu.ac.jp (J.T.); 3 Division of Antiviral Therapy, Joint Research Center for Human Retrovirus Infection, Kagoshima University, 1-21-24 Korimoto, Kagoshima City 890-8544, Kagoshima, Japan; yakahori@kufm.kagoshima-u.ac.jp (Y.A.); kmaeda@kufm.kagoshima-u.ac.jp (K.M.); 4 Department of Virology II, National Institute of Infectious Diseases, Japan Institute for Health Security (JIHS), 1-23-1 Toyama, Shinjuku-ku, Tokyo 162-8640, Japan; m-toyama@niid.go.jp; 5 Faculty of Pharmaceutical Sciences, Tokyo University of Science, 6-3-1 Niijuku, Katsushika-ku, Tokyo 125-8585, Japan; yasumoto@rs.tus.ac.jp; 6 Tropical Technology Plus, 12-75 Suzaki, Uruma City 904-2234, Okinawa, Japan; hirose_fweu@cs.u-ryukyu.ac.jp; 7 Influenza Research Center, National Institute of Infectious Diseases, Japan Institute for Health Security, 4-7-1 Gakuen, Musashimurayama City 208-0011, Japan; keim@niid.go.jp

**Keywords:** onnamides A and B, hepatitis B virus, viral transcription inhibition

## Abstract

We recently reported that onnamide A, a marine-derived natural compound isolated from the sponge *Theonella* sp., inhibits the entry process of SARS-CoV-2 infection. However, its antiviral activity against other viruses remains largely unexplored. Here, we investigated the effects of onnamide A and its structurally related analog, onnamide B, on hepatitis B virus (HBV) infection. Using iNTCP cells, a hepatoblastoma-derived cell line permissive to HBV infection, we found that onnamides A and B exhibited cytotoxicity, with CC_50_ values of 0.53 ± 0.10 μM and 2.37 ± 0.25 μM, respectively. Following HBV infection, the levels of total HBV RNA were significantly reduced by onnamide A (IC_50_ = 0.06 ± 0.01 μM) and onnamide B (IC_50_ = 0.23 ± 0.06 μM). Notably, both compounds markedly decreased the levels of HBV pregenomic RNA. Furthermore, significant inhibition was particularly evident when onnamide treatment was initiated after HBV infection. Consistent with these observations, onnamides did not affect HBV binding, entry, or covalently closed circular DNA formation, but they significantly suppressed HBV RNA transcription. In particular, the transcriptional activities driven by the core and X promoters were markedly inhibited by onnamide treatment. Taken together, our findings demonstrate that onnamides possess potent anti-HBV activity and highlight their potential as candidate compounds targeting HBV RNA transcription.

## 1. Introduction

Chronic HBV infection remains a major global health burden, affecting an estimated 254 million individuals worldwide [1]. Current therapeutic options for chronic HBV infection mainly consist of nucleos(t)ide analogs, such as tenofovir [2], which potently inhibit viral reverse transcription, and pegylated interferon-α, which enhances host immune responses. Although these treatments effectively suppress viral replication and improve clinical outcomes, they rarely eliminate HBV covalently closed circular DNA (cccDNA) or achieve a functional cure, often necessitating lifelong therapy [3]. Therefore, there is an urgent need to develop novel therapeutic agents that target different stages of the HBV life cycle or act through distinct mechanisms, ultimately aiming for a complete or functional cure for chronic HBV infection.

HBV entry into hepatocytes occurs through a specific interaction with the sodium taurocholate co-transporting polypeptide (NTCP) receptor [4]. After entry, the viral capsid is transported to the nucleus, where uncoating occurs and the relaxed circular DNA is converted into cccDNA. The HBV genome, which exists as cccDNA in the nucleus, serves as the transcriptional template for five species of HBV mRNAs with overlapping 3′ ends: the 3.5-kb pregenomic RNA (pgRNA) and precore mRNA (pcRNA), the 2.4-kb preS1 mRNA, the 2.1-kb preS2/S mRNA, and the 0.7-kb X mRNA. These transcripts are regulated by the core, preS1, preS2/S, and X promoters, respectively [5]. Among them, pgRNA plays a crucial role as the template for reverse transcription during viral replication. The pgRNA is encapsidated together with the viral polymerase into nucleocapsids, where reverse transcription occurs, producing new viral DNA that is subsequently assembled into progeny virions and secreted. Consequently, inhibition of pgRNA expression represents a promising strategy for the development of curative therapies for chronic HBV infection. Several HBV core promoter inhibitors, including HX531, calcitriol, and MafF, have been identified, although most remain in the preclinical stage [6,7,8,9].

Natural products derived from marine organisms have long been recognized as valuable sources of structurally diverse and biologically active compounds, many of which have inspired the development of modern pharmaceuticals [10]. Marine environments provide unique ecological conditions that promote the biosynthesis of secondary metabolites with distinctive chemical scaffolds and potent biological activities. In particular, the marine-derived metabolites have yielded promising antiviral agents against viruses such as human immunodeficiency virus, influenza, and coronaviruses, demonstrating their potential for broad-spectrum antiviral drug discovery [11,12,13].

Onnamide A, a marine-derived compound isolated from the sponge *Theonella* sp. [14], has been reported to exhibit cytotoxic and protein synthesis inhibitory activities [15,16]. Recently, we demonstrated that onnamide A inhibits the entry process of SARS-CoV-2 infection, underscoring its potential as a broad-spectrum antiviral agent [17]. In addition, onnamides have shown antiprotozoal activity against the promastigote form of *Leishmania major* [18]. However, their activity against other viruses, including HBV, has not been thoroughly investigated.

In this study, we investigated the antiviral effects of onnamide A and its structural analog, onnamide B, against HBV infection using HBV-susceptible HepG2 cells with a doxycycline-inducible NTCP gene (iNTCP cells) [19]. We assessed their cytotoxicity, effects on total HBV RNA, and particularly their ability to suppress pgRNA expression. Notably, the transcriptional activities driven by the core and X promoters were markedly inhibited by onnamide treatment. These findings provide new insights into the antiviral potential of onnamides and suggest their promise as lead compounds for the development of novel anti-HBV therapies.

## 2. Results

### 2.1. Onnamides A and B, Marine-Derived Natural Compounds Isolated from the Sponge Theonella sp.

**Figure 1:**
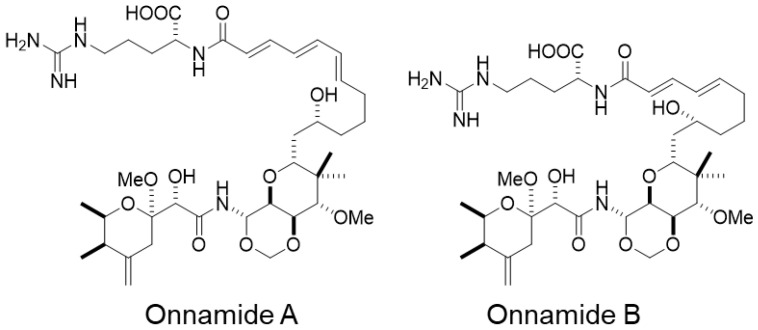
Structures of onnamides. Onnamides A and B share a similar polyketide-derived core structure but differ in the substitution pattern of the side chain. Specifically, onnamide B lacks one hydroxyl group present in onnamide A, resulting in a slight difference in polarity and potentially in bioactivity.

We previously reported that onnamide A, a marine-derived natural product isolated from the sponge *Theonella* sp., inhibits the entry of SARS-CoV-2 [17]. However, its antiviral effects against other viruses have not been fully elucidated. This study aimed to evaluate the anti-HBV activity of onnamides A and B (Figure 1), two structurally related analogs belonging to the onnamide family.

### 2.2. Onnamides A and B Suppress the Levels of Total HBV RNA and pgRNA/pcRNA

**Figure 2:**
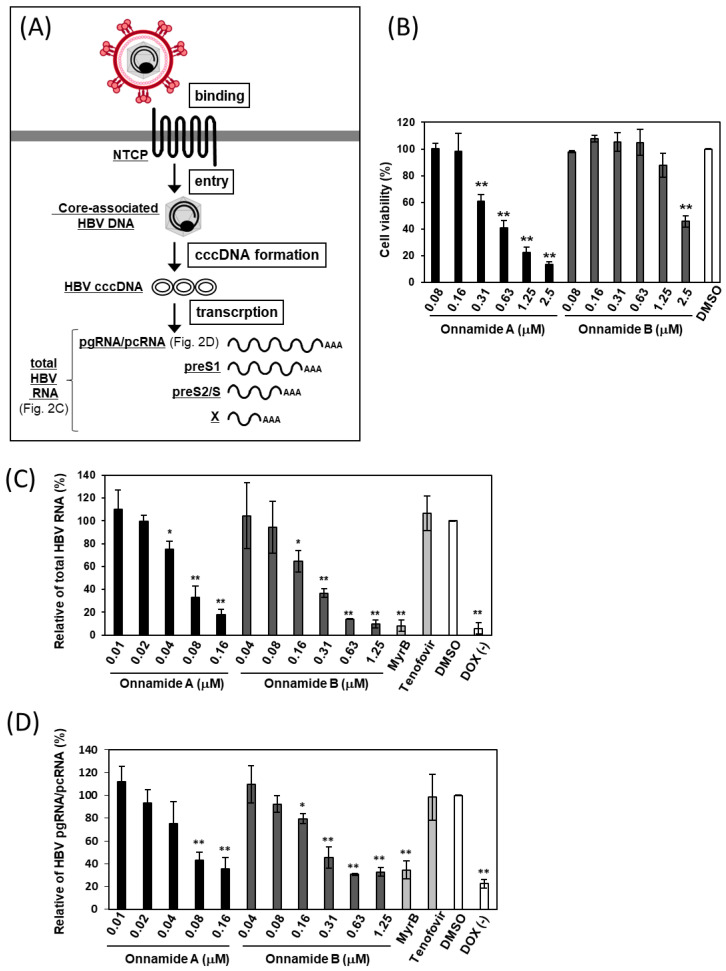
Effects of onnamides A and B on cell viability, total HBV RNA, and pgRNA/pcRNA. (**A**) Schematic representation of the HBV life cycle, illustrating the steps from viral binding to transcription. The effects of onnamides A and B on cell viability (**B**), total HBV RNA (**C**), and pgRNA/pcRNA (**D**) were examined in iNTCP cells treated with the indicated concentrations of the compounds. (**B**) Cell viability was assessed using the WST-8 assay and is shown as % inhibition relative to DMSO. Data represent mean ± SD (*n* = 3). (**C**,**D**) Total HBV RNA (**C**) and HBV pgRNA/pcRNA (**D**) were quantified by RT-qPCR. MyrB (0.5 μM), an entry inhibitor, and tenofovir (1 μM), a reverse transcriptase inhibitor, were included as control drugs. Results are shown as % inhibition relative to DMSO. Data represent mean ± SD (*n* = 3). Asterisks indicate significance levels (* *p* < 0.05, ** *p* < 0.01).

To investigate whether onnamides exhibit anti-HBV activity through a mechanism distinct from that of the existing reverse transcriptase inhibitor tenofovir [2], we focused on the early phase of HBV infection, from viral entry to transcription (Figure 2A). Experiments were conducted using iNTCP cells, in which expression of the HBV receptor NTCP is inducible by doxycycline (DOX) [19].

We first determined the non-toxic concentrations of onnamides in iNTCP cells (Figure 2B). After a 3-day exposure to various concentrations, onnamides A and B exhibited cytotoxicity at concentrations ≥ 0.31 µM and ≥2.5 µM, with CC_50_ values of 0.53 ± 0.10 μM and 2.37 ± 0.25 μM, respectively. Based on these results, we next evaluated the effects of onnamides on HBV infection at non-toxic concentrations (Figure 2C,D). As shown in Figure 2C,D, DOX-mediated induction of NTCP expression led to a significant increase in total HBV RNA and pgRNA/pcRNA compared with DOX-untreated cells. Furthermore, Myrcludex B (MyrB), a preS1-derived peptide and competitive inhibitor of HBV binding [20], significantly suppressed the levels of total HBV RNA and pgRNA/pcRNA, whereas tenofovir did not, indicating that this NTCP-dependent assay specifically evaluates the early phase of HBV infection. As shown in Figure 2C, compared with DMSO-treated control cells, onnamide A markedly reduced the levels of total HBV RNA, including pgRNA/pcRNA, preS1, preS2/S, and X RNAs, at concentrations ≥ 0.04 µM, with an IC_50_ value of 0.06 ± 0.01 μM. Similarly, onnamide B significantly decreased total HBV RNA levels at concentrations ≥ 0.16 µM, with an IC_50_ value of 0.23 ± 0.06 μM. The selectivity indices (SI) for onnamides A and B were calculated to be 8.83 and 10.30, respectively. Furthermore, intracellular levels of HBV pgRNA were significantly reduced by onnamide A at concentrations ≥ 0.08 µM, and by onnamide B at concentrations ≥ 0.16 µM (Figure 2D).

### 2.3. Onnamides A and B Most Effectively Suppress HBV at a Post-Entry Stage of the Viral Life Cycle

**Figure 3:**
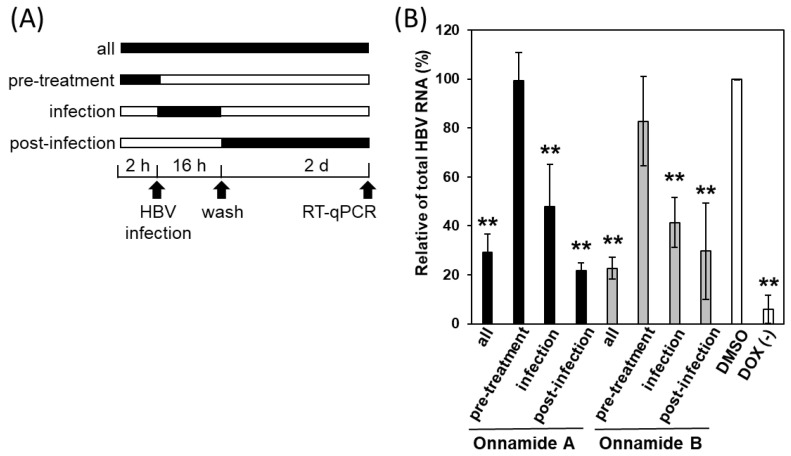
Onnamides A and B inhibit HBV most effectively at a post-entry stage of the viral life cycle. (**A**) Time-of-addition experiments with the compounds. iNTCP cells were treated with the compounds before, during, or after HBV infection, as illustrated in the schematic. (**B**) iNTCP cells were treated with onnamide A (0.16 μM) or onnamide B (0.63 μM). Total HBV RNA was quantified by RT-qPCR and is shown as % inhibition relative to DMSO. Data represent mean ± SD (*n* = 3). Asterisks indicate significance levels (** *p* < 0.01).

Next, to broadly examine the stage of the HBV life cycle affected by onnamides A and B, we performed time-of-addition experiments in iNTCP cells. Cells were treated with the compounds either before, during, or after HBV infection, and the levels of total HBV RNA were quantified by RT-qPCR (Figure 3A).

We first validated our time-of-addition assay using control compounds in iNTCP cells (Supplementary Figure S1). The binding inhibitor MyrB suppressed infection when applied both as a pre-treatment and during infection, whereas troglitazone [21], which inhibits entry by blocking internalization, suppressed infection only when administered during infection. The transcription inhibitor HX531 [6,7] inhibited infection when applied during and post-infection. These results confirm that this assay can effectively distinguish antiviral activities at different stages of the HBV life cycle.

Using this system, we then evaluated onnamides A and B. Little to no antiviral activity was observed when they were administered prior to infection, whereas significant inhibition of total HBV RNA was observed when treatment was initiated at the time of infection or post-infection, with the latter most effectively reducing HBV RNA (Figure 3B). These results suggest that the antiviral activity of onnamides A and B occurs at a post-entry stage of the HBV life cycle.

### 2.4. Onnamides A and B Suppress HBV RNA Transcription

**Figure 4:**
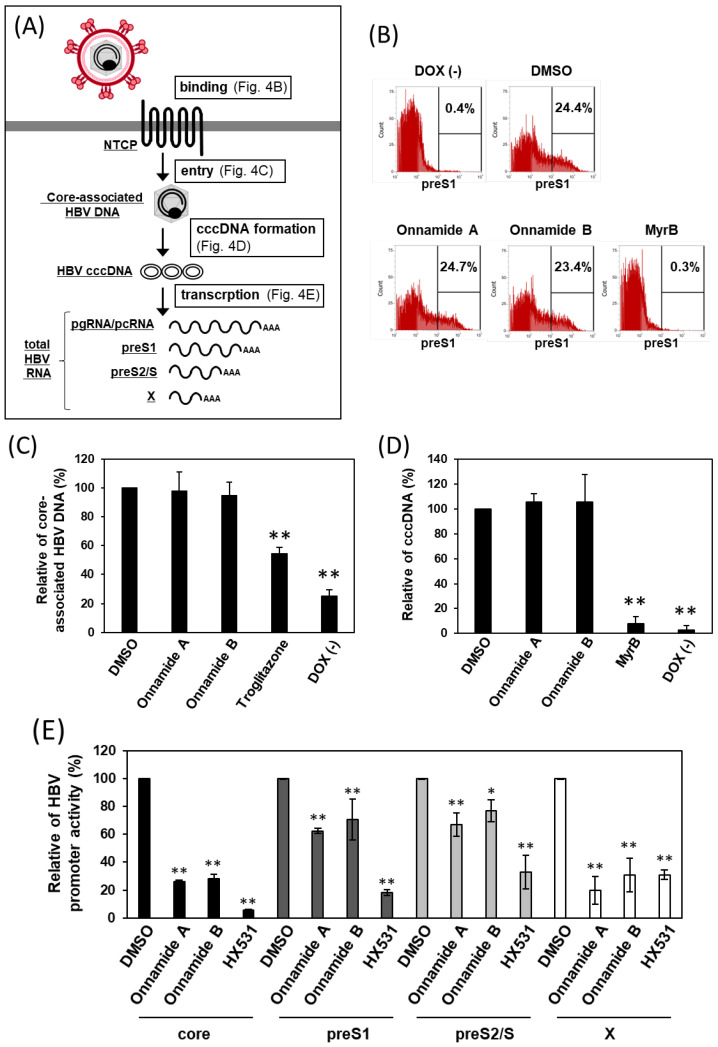
Onnamide A and B suppress HBV RNA transcription. (**A**) The effects of onnamides A and B on HBV binding (**B**), entry (**C**), cccDNA formation (**D**), and transcription (**E**) were analyzed as illustrated in the schematic. (**B**) HBV preS1-mediated binding to host cells was examined in iNTCP cells treated with onnamide A (0.16 μM), onnamide B (0.63 μM), or MyrB (0.5 μM), followed by incubation with a TAMRA-labeled preS1 peptide. Cellular binding of the fluorescently labeled preS1 peptide was analyzed by flow cytometry. Ten thousand cells were measured in each sample. The percentage of cells within the indicated fluorescence range is shown. Data are from a single representative experiment; similar results were obtained in three independent experiments. (**C**) iNTCP cells were attached with HBV at 4 °C, then shifted to 37 °C for internalization in the presence of onnamide A (0.16 µM), onnamide B (0.63 µM), or troglitazone (50 µM). Core-associated HBV DNA was quantified by RT-qPCR and expressed as % inhibition relative to DMSO. Data represent mean ± SD (*n* = 3). (**D**) iNTCP cells were treated with onnamide A (0.16 µM), onnamide B (0.63 µM), or MyrB (0.5 µM) before HBV infection. HBV cccDNA levels were measured by RT-qPCR and are shown as % inhibition relative to DMSO. Data represent mean ± SD (*n* = 3). (**E**) iNTCP cells transfected with HBV promoter-driven luciferase reporters were treated with onnamide A (0.16 µM), onnamide B (0.63 µM), or HX531 (5 µM). Firefly luciferase activity was normalized to Renilla. Data represent mean ± SD (*n* = 3). Asterisks indicate significance levels (* *p* < 0.05, ** *p* < 0.01).

We next examined in more detail the stage of the HBV life cycle targeted by onnamides (Figure 4A). First, to assess the effect of onnamides on HBV preS1-mediated binding to target cells, we used C-terminally 5-carboxytetramethylrhodamine (TAMRA)-conjugated and N-terminally myristoylated preS1 peptides corresponding to amino acids 2–48 (preS1-TAMRA), which serve as an HBV mimic (Figure 4B). In cells in which NTCP expression was induced by DOX treatment, the proportion of preS1-binding cells increased compared with DOX-untreated cells, and treatment with the binding inhibitor MyrB reduced this proportion. Onnamide-treated cells showed a proportion of preS1-binding cells comparable to that of DMSO-treated cells, indicating that onnamides do not affect HBV attachment.

Next, we examined the effect of onnamides on HBV entry (Figure 4C) and cccDNA formation (Figure 4D). As shown in Figure 4C, treatment with troglitazone, an internalization inhibitor, significantly reduced core-associated HBV DNA compared with DMSO-treated cells, whereas onnamide treatment resulted in HBV DNA levels comparable to those in DMSO-treated cells. As shown in Figure 4D, intracellular HBV cccDNA levels were also unaffected by onnamide treatment. Taken together, these findings demonstrate that onnamides A and B do not affect viral binding, entry, or cccDNA formation.

We then performed an HBV promoter luciferase assay to evaluate the impact of onnamides on viral transcriptional regulation. Onnamide treatment significantly reduced the activities of all HBV promoters examined, including the core, preS1, preS2/S2, and X promoters, indicating a broad suppressive effect on HBV transcriptional machinery. Notably, the activities of the core and X promoters were suppressed by more than 70% compared with DMSO-treated cells, suggesting that these promoters are particularly sensitive to onnamide-mediated inhibition. Given that the core promoter governs the production of pgRNA/pcRNA, and the X promoter drives HBx expression—both of which are essential for efficient viral replication—these findings imply that onnamides may strongly interfere with key transcriptional steps required for HBV propagation. Taken together, these results demonstrate that onnamides exert potent inhibitory effects on multiple HBV promoters, with especially pronounced suppression of core and X promoter activity.

## 3. Discussion

**Figure 5:**
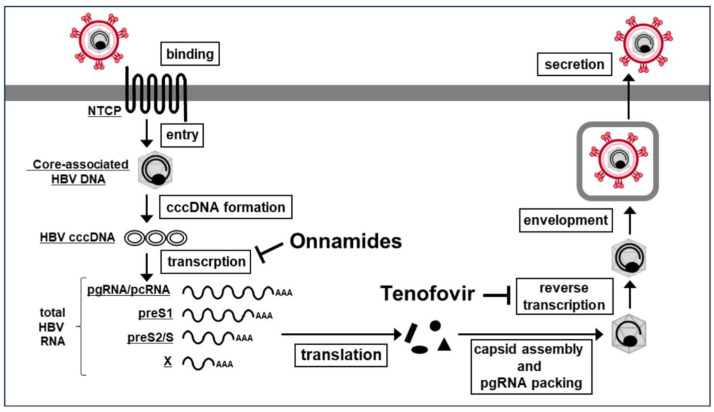
Onnamides A and B suppress HBV RNA transcription through a mechanism distinct from the reverse transcriptase inhibitor tenofovir.

In this study, we demonstrated that the marine-derived natural compounds onnamides A and B exhibit potent antiviral activity against HBV through a mechanism clearly distinct from that of the clinically used reverse transcriptase inhibitor tenofovir (Figure 5). Whereas tenofovir suppresses HBV replication by inhibiting reverse transcription, it showed no effect in the NTCP-dependent early-phase infection assay used here. In contrast, onnamides markedly reduced HBV RNA levels in this system, indicating that they act at a step upstream of reverse transcription and thus suppress HBV through an entirely different antiviral mechanism. Onnamide A was previously shown to partially inhibit SARS-CoV-2 entry [17], but our HBV data reveal a distinct antiviral profile. Onnamides did not affect preS1-mediated HBV binding or internalization, indicating that their anti-HBV activity, unlike their anti–SARS-CoV-2 activity, is independent of viral entry inhibition. Time-of-addition experiments further confirmed that onnamides exert their antiviral effects only when added at the time of or after infection, consistent with targeting intracellular post-entry events.

Mechanistically, onnamides suppressed HBV RNA synthesis, including total viral RNA as well as pgRNA/pcRNA, at non-toxic concentrations. Interestingly, the most potent inhibitory effects were observed for the core and X promoters. Although the exact molecular target(s) of onnamides remain unknown, previous studies have reported that onnamide A induces ribotoxic stress through inhibition of protein synthesis, thereby activating p38 mitogen-activated protein kinase (MAPK) and c-Jun N-terminal kinase (JNK), and triggering apoptosis [16]. Notably, inhibition of p38 MAPK has been reported to suppress HBV replication: treatment with the p38 MAPK-specific inhibitor SB203580 reduced levels of HBV DNA in human hepatoma cells [22]. Similarly, suppression of JNK activity by chlorogenic acid has been shown to inhibit HBV replication [23]. In contrast, onnamide A was reported to activate both p38 MAPK and JNK [16], suggesting that the antiviral effect observed in this study is unlikely to be mediated through simple activation of these stress kinases. Thus, while onnamide-induced ribotoxic stress activates p38 MAPK and JNK pathways in other cellular contexts, the mechanism by which onnamide A suppresses HBV core and X promoter activity appears to be distinct from these kinase-dependent pathways and may involve alternative transcriptional or stress-responsive processes.

Several mechanisms may explain why the core promoter is more sensitive to onnamide-mediated suppression. The core promoter is among the most transcriptionally active regulatory elements in the HBV genome and governs the production of pgRNA/precore mRNA, both required for nucleocapsid formation and viral replication. Because core promoter activity depends on a complex network of hepatocyte-enriched transcription factors, including HNF4α, C/EBP, and RXRα [24,25,26], even modest perturbations in host transcriptional programs may disproportionately impact its activity. This high dependence on multiple transcriptional regulators may make the core promoter particularly vulnerable to transcriptional stress induced by onnamide. Further investigation into their mechanism of action, including identification of molecular targets and in vivo efficacy studies, will be critical to evaluate their feasibility as therapeutic agents for chronic HBV infection.

Collectively, these findings reveal that onnamides A and B suppress HBV replication by inhibiting viral transcription downstream of entry, through a mechanism distinct not only from their antiviral activity against SARS-CoV-2 but also from existing HBV therapeutics such as tenofovir. This unique transcription-targeting activity highlights the potential of onnamides as promising leads for developing novel HBV antiviral agents and as valuable research tools for dissecting the transcriptional regulation of HBV cccDNA.

## 4. Materials and Methods

### 4.1. Specimens, Extraction, and Isolation of Onnamides A and B

Onnamides A (yield: 110.5 mg) and B (13.5 mg) were isolated from fresh Okinawan sponges of the genus *Theonella* (wet weight: 6.5 kg), collected at Manza, Okinawa, Japan (26°29′36.0″ N, 127°50′02.9″ E) on 26 June 2024. The detailed isolation procedure was described in our previous work [18]. The onnamides were identified by comparing their NMR data (500 MHz, Bruker Avance III 500 spectrometer, Bruker, Billerica, MA, USA) and Orbitrap Exploris 240 (Thermo Fisher Scientific, Waltham, MA, USA) data with those reported previously [14].

### 4.2. Cells and HBV Preparation

iNTCP cells were kindly provided by Dr. Kei Miyakawa and Dr. Akihide Ryo (Japan Institute for Health Security, Japan). iNTCP cells were maintained in DMEM supplemented with 10% fetal bovine serum (FBS), 100 µg/mL penicillin, 100 µg/mL streptomycin, and 1 µg/mL puromycin as previously described [19]. The HBV (genotype D) used as the inoculum was collected from the culture supernatant of Hep38.7-Tet cells grown in the absence of tetracycline, filtered through a 0.45-μm membrane, and concentrated approximately 100-fold using polyethylene glycol 8000 (PEG8000) [7].

### 4.3. Cytotoxicity Assay

iNTCP cells were seeded into 96-well plates at a density of 1 × 10^4^ cells per well. After 24 h, the cells were treated with 10 μg/mL Dox. On the following day, the cells were incubated with onnamide in the presence of 10 μg/mL Dox, 4% PEG8000, and 2% dimethyl sulfoxide (DMSO) for 3 days. Cytotoxicity was assessed using the WST-8 assay with the Cell Counting Kit-8 (Dojindo Laboratories, Kumamoto, Japan), and the 50% cytotoxic concentration (CC_50_) was determined.

### 4.4. Quantification of Total HBV RNA and pgRNA/pcRNA by RT-qPCR

The HBV infectivity assay using RT-qPCR was conducted as previously described with slight modifications [4,27]. iNTCP cells were seeded in 24-well plates (6 × 10^4^ cells/well). After 24 h, the cells were treated with 10 μg/mL Dox. The following day, cells were cultured with onnamide and, subsequently, HBV at 8000 genome equivalents (GEq)/cell in the presence of 10 μg/mL Dox, 4% PEG8000, and 2% DMSO. After 3 days, the cells were washed with phosphate-buffered saline (PBS) and harvested, and viral RNA was extracted using ISOSPIN Cell & Tissue RNA (Nippon Gene, Tokyo, Japan). Subsequently, quantitative RT-qPCR was performed using One Step TB Green^®^ Prime-Script™ RT-PCR Kit II (Takara Bio Inc., Shiga, Japan) according to the manufacturer’s instructions. The primer pairs used were 5′-GCACTTCGCTTCACCTCTGC-3′ and 5′-CTCAAGGTCGGTCGTTGACA-3′ for total HBV RNA; 5′-GAGTGTGGATTCGCACTCC -3′ and 5′-GAGGCGAGGGAGTTCTTCT-3′ for HBV pgRNA/pcRNA; 5′-GCACCGTCAAGGCTGAGAAC-3′ and 5′-GGATCTCGCTCCTGGAAGATG-3′ for GAPDH as internal control.

### 4.5. Time-of-Addition Assay

Time-of-addition assay was conducted as previously described with slight modifications [7]. iNTCP cells were seeded in 24-well plates (6 × 10^4^ cells/well). After 24 h, the cells were treated with 10 μg/mL Dox. The following day, the cells were treated with onnamide A (0.16 μM) or onnamide B (0.63 μM) before, during, or after HBV inoculation at 8000 GEq/cell. For the pre-treatment condition, cells were incubated with the compound for 2 h prior to HBV infection and the compound was removed before viral inoculation. For the co-treatment condition, the compound was added simultaneously with HBV during the inoculation period for 16 h. For the post-treatment condition, the compound was added immediately after the inoculum was removed and maintained throughout the culture period for 2 days. As described above, cells were harvested, followed by RNA extraction and quantification of total HBV RNA by RT-qPCR.

### 4.6. preS1 Binding Assay

HBV preS1 binding assay was conducted as previously described with slight modifications [28]. The preS1-TAMRA were synthesized by Scrum Inc. (Tokyo, Japan). iNTCP cells were seeded in 24-well plates (6 × 10^4^ cells/well). After 24 h, the cells were treated with 10 μg/mL Dox. The following day, the cells were detached and adjusted to 5 × 10^4^ cells/tube. The cells were then treated with onnamide A (0.16 μM), onnamide B (0.63 μM), or MyrB (0.5 μM) for 1 h at 37 °C, followed by incubation with preS1-TAMRA (0.25 μM) for 1 h at 4 °C. After washing three times with PBS, the binding of preS1-TAMRA to the cells was quantified using an Attune™ NxT Flow Cytometer (Thermo Fisher Scientific).

### 4.7. Quantification of Core-Associated HBV DNA by RT-qPCR

Core-associated HBV DNA was extracted and quantified as previously described, with slight modifications [21,29,30]. iNTCP cells were seeded in 6-well plates at a density of 5 × 10^5^ cells per well. After 24 h, the cells were treated with 10 μg/mL Dox. On the following day, the cells were infected with HBV at 8000 GEq/cell in the presence of 10 μg/mL Dox, 4% PEG8000, and 2% DMSO at 4 °C for 2 h. The cells were then washed thoroughly with cold medium before shifting the temperature to 37 °C to allow viral internalization in the presence of onnamide A (0.16 μM), onnamide B (0.63 μM), or troglitazone (50 μM). After 6 h, the cells were treated with trypsin for 5 min at 37 °C. Complete medium was subsequently added to detach the cells, which were collected into a tube. Cells were centrifuged at 2000× *g* for 5 min at 4 °C, the supernatant was removed, and the cell pellet was washed with PBS containing 5 mM EDTA, followed by a second centrifugation at 2000× *g* for 5 min at 4 °C. The supernatant was then removed.

The cell pellet was then treated with lysis buffer (50 mM Tris-HCl [pH 7.5], 1 mM EDTA, 150 mM NaCl, and 1% NP-40) and incubated for 10 min at 37 °C. Nuclei and cellular debris were removed by centrifugation. The resulting cell lysates were treated with 6 mM magnesium acetate, DNase I (100 U/mL), and RNase A (0.2 mg/mL) for 2 h at 37 °C to remove unencapsidated nucleic acids. The reaction was stopped by adding 10 mM EDTA and incubating for 15 min at 65 °C. Subsequently, the lysates were treated with proteinase K (0.2 mg/mL) and 1% SDS and incubated for 2 h at 37 °C. Intracellular core-associated HBV DNA was then purified by phenol–chloroform–isoamyl alcohol (25:24:1) extraction, followed by DNA precipitation with isopropanol. The DNA pellet was collected by centrifugation at 15,000× *g* for 15 min at 4 °C, washed with 70% ethanol, air-dried, and resuspended in 30 μL nuclease-free water.

Quantitative RT-qPCR was subsequently performed using TB Green^®^ Premix Ex Taq™ II (Takara Bio) according to the manufacturer’s instructions. The primer pairs used were 5′-AAGGTAGGAGCTGGAGCATTCG-3′ and 5′-AGGCGGATTTGCTGGCAAAG-3′ for HBV DNA, and 5′-GCACACTCATCACAGCGCTAA-3′ and 5′-GATTATGGATGCGGTTGCTTG-3′ for mitochondrial DNA, which was used as an internal control.

### 4.8. Quantification of HBV cccDNA by RT-qPCR

HBV cccDNA was extracted and quantified as previously described, with slight modifications [31,32,33]. iNTCP cells were seeded in 6-well plates (5 × 10^5^ cells/well). After 24 h, the cells were treated with 10 μg/mL Dox. The following day, cells were treated with onnamide A (0.16 µM), onnamide B (0.63 µM), or MyrB (0.5 µM) and subsequently infected with HBV at 8,000 GEq/cell in the presence of 10 μg/mL Dox, 4% PEG8000, and 2% DMSO.

After 3 days, the cells were treated with trypsin for 5 min at 37 °C. Complete medium was then added to detach the cells, which were collected into a tube. The cells were centrifuged at 2000× *g* for 5 min at 4 °C, the supernatant was removed, and the cell pellet was washed with PBS and centrifuged again at 2000× *g* for 5 min at 4 °C. The supernatant was removed, and the cell pellet was treated with 0.6% sodium dodecyl sulfate and 10 mM Tris-HCl (pH 7.5) at room temperature for 30 min. Sodium chloride was then added to a final concentration of 1 M, followed by vortexing, and the samples were incubated at 4 °C overnight. The next day, the samples were centrifuged at 15,000× *g* at 4 °C for 30 min, and the supernatant was collected. DNA was extracted using phenol–chloroform–isoamyl alcohol (25:24:1), followed by DNA precipitation with isopropanol, and the DNA pellet was collected by centrifugation. The DNA was then treated with T5 exonuclease (0.2 U/µL) at 37 °C for 1 h, followed by phenol–chloroform–isoamyl alcohol extraction and isopropanol precipitation. The final DNA pellet was collected by centrifugation and resuspended in 30 μL of nuclease-free water.

Quantitative RT-qPCR was subsequently performed using THUNDERBIRD^®^ Probe qPCR Mix (TOYOBO, Osaka, Japan) according to the manufacturer’s instructions. The primer pairs used for HBV cccDNA were 5′-CCGTGTGCACTTCGCTTCA-3′ and 5′-GCACAGCTTGGAGGCTTGA-3′, and the probe sequence was 5′-FAM-CATGGAGACCACCGTGAACGCCC-TAMRA-3′. Mitochondrial DNA was used as an internal control.

### 4.9. HBV Promoter Activity Measurement Using Dual-Luciferase Reporter System

The promoter regions of HBV core, preS1, preS2/S, and X were amplified by PCR [34] from the DNA of HepG2.2.15 cells as a template, which continuously express HBV owing to stable integration of the viral genome, using the following primers (underlined sequences indicate KpnI sites, and double-underlined sequences indicate HindIII sites): core promoter: 5′-GGCCGGTACCGGTCTTACATAAGAGGAC-3′ and 5′-TGCCAAGCTTTGAACAAGAGATGATTAG-3′, preS1 promoter: 5′-GGCCGGTACCCTCACTTTTGGAAGAGAAA-3′ and 5′-TGCCAAGCTTCTTATATAATATACCCGC-3′, preS2/S promoter:5′-GGCCGGTACCGTGGGTCACCATATTCTTG-3′ and 5′-TGCCAAGCTTCTTCCTGTCTGGCGATTG-3′, X promoter: 5′-GGCCGGTACCTGCGTGGAACCTTTTCGG-3′ and 5′-TGCCAAGCTTTGGATACGATGTATATTTG-3′. These PCR products were subsequently cloned into the pGL4.16[luc2CP/Hygro] vector (Promega, Madison, WI, USA) to generate firefly luciferase reporter plasmids under the control of the respective HBV promoters.

iNTCP cells were co-transfected with the firefly luciferase reporter plasmid and a Renilla luciferase plasmid (pRL-CMV, Promega), under the control of the cytomegalovirus promoter, using Lipofectamine 3000 Transfection Reagent (Thermo Fisher Scientific). At 6 h post-transfection, cells were treated with onnamide A (0.16 μM), onnamide B (0.63 μM), or HX531 (5 μM) for an additional 24 h. Luciferase activity was measured using a Dual-Luciferase Reporter Assay System (Promega). Firefly and Renilla luciferase activities were detected using a GloMax Explorer luminometer (Promega), and firefly luciferase activity was normalized to Renilla luciferase activity.

### 4.10. Statistical Analysis

All statistical analyses were performed using GraphPad Prism 6 (GraphPad Software, San Diego, CA, USA). For comparisons, we conducted a one-way ANOVA followed by Dunnett’s test. The results are presented as the mean ± SD. *p* < 0.05 was considered statistically significant. Asterisks indicate significance levels (* *p* < 0.05, ** *p* < 0.01).

## 5. Conclusions

Onnamides A and B exhibit potent anti-HBV activity by specifically suppressing HBV RNA transcription without affecting viral entry or cccDNA formation. These findings highlight their potential as candidate compounds targeting HBV transcription for therapeutic development.

## Data Availability

All data supporting the findings of this study are contained within the article and its Supplementary Materials.

## Supplementary Materials

The following supporting information can be downloaded at: https://www.mdpi.com/article/10.3390/md24010021/s1, Supplementary Figure S1: Time-of-addition experiments with the control compounds in iNTCP cells.

## Abbreviations

The following abbreviations are used in this manuscript:

cccDNA	covalently closed circular DNA
DMSO	dimethyl sulfoxide
DOX	doxycycline
FBS	fetal bovine serum
GEq	genome equivalents
HBV	hepatitis B virus
iNTCP	HBV-susceptible HepG2 cells with a doxycycline-inducible NTCP gene
JNK	c-Jun N-terminal kinase
MAPK	mitogen-activated protein kinase
MyrB	Myrcludex B
NTCP	the sodium taurocholate co-transporting polypeptide
PBS	phosphate-buffered saline
pcRNA	precore mRNA
PEG8000	polyethylene glycol 8000
pgRNA	pregenomic RNA
preS1-TAMRA	C-terminally 5-carboxytetramethylrhodamine-conjugated and N-terminally myristoylated preS1 peptides corresponding to amino acids 2–48
SI	selectivity index

## References

[1] World Health Organization Hepatitis B. https://www.who.int/news-room/fact-sheets/detail/hepatitis-b.

[2] Lou L. (2013). Advances in nucleotide antiviral development from scientific discovery to clinical applications: Tenofovir disoproxil fumarate for hepatitis B. J. Clin. Transl. Hepatol..

[3] Boonstra A., Sari G. (2025). HBV cccDNA: The Molecular Reservoir of Hepatitis B Persistence and Challenges to Achieve Viral Eradication. Biomolecules.

[4] Yan H., Zhong G., Xu G., He W., Jing Z., Gao Z., Huang Y., Qi Y., Peng B., Wang H. (2012). Sodium taurocholate cotransporting polypeptide is a functional receptor for human hepatitis B and D virus. eLife.

[5] Peng B., Pan L., Li W. (2024). New Insights on Hepatitis B Virus Viral Transcription in Single Hepatocytes. Viruses.

[6] Raney A.K., Johnson J.L., Palmer C.N., McLachlan A. (1997). Members of the nuclear receptor superfamily regulate transcription from the hepatitis B virus nucleocapsid promoter. J. Virol..

[7] Watashi K., Liang G., Iwamoto M., Marusawa H., Uchida N., Daito T., Kitamura K., Muramatsu M., Ohashi H., Kiyohara T. (2013). Interleukin-1 and Tumor Necrosis Factor-α trigger restriction of hepatitis B virus infection via a cytidine deaminase activation-induced cytidine deaminase (AID). J. Biol. Chem..

[8] Ahluwalia S., Choudhary D., Tyagi P., Kumar V., Vivekanandan P. (2021). Vitamin D signaling inhibits HBV activity by directly targeting the HBV core promoter. J. Biol. Chem..

[9] Ibrahim M.K., Abdelhafez T.H., Takeuchi J.S., Wakae K., Sugiyama M., Tsuge M., Ito M., Watashi K., El Kassas M., Kato T. (2021). MafF is an antiviral host factor that suppresses transcription from hepatitis B virus core promoter. J. Virol..

[10] Carroll A.R., Copp B.R., Grkovic T., Keyzers R.A., Prinsep M.R. (2024). Marine natural products. Nat. Prod. Rep..

[11] Lu Z., Van Wagoner R.M., Harper M.K., Baker H.L., Hooper J.N.A., Bewley C.A., Ireland C.M. (2011). Mirabamides E–H, HIV-Inhibitory Depsipeptides from the Sponge *Stelletta clavosa*. J. Nat. Prod..

[12] Chen Y.-H., Hsieh C.-Y., Chiou C.-T., Caro E.J.G.V., Tayo L.L., Tsai P.-W. (2025). In Vitro and In Silico Studies on the Anti-H1N1 Activity of Bioactive Compounds from Marine-Derived *Streptomyces ardesiacus*. Mar. Drugs.

[13] Surti M., Patel M., Adnan M., Moin A., Ashraf S.A., Siddiqui A.J., Snoussi M., Deshpande S., Reddy M.N. (2020). Ilimaquinone (marine sponge metabolite) as a novel inhibitor of SARS-CoV-2 key target proteins in comparison with suggested COVID-19 drugs: Designing, docking and molecular dynamics simulation study. RSC Adv..

[14] Sakemi S., Ichiba T., Kohmoto S., Saucy G., Higa T. (1988). Isolation and structure elucidation of onnamide A, a new bioactive metabolite of a marine sponge, Theonella sp.. J. Am. Chem. Soc..

[15] Burres N.S., Clement J.J. (1989). Antitumor activity and mechanism of action of the novel marine natural products mycalamide-A and -B and onnamide. Cancer Res..

[16] Lee K.-H., Nishimura S., Matsunaga S., Fusetani N., Horinouchi S., Yoshida M. (2005). Inhibition of protein synthesis and activation of stress-activated protein kinases by onnamide A and theopederin B, antitumor marine natural products. Cancer Sci..

[17] Hayashi Y., Higa N., Yoshida T., Tyas T.A., Mori-Yasumoto K., Yasumoto-Hirose M., Tani H., Tanaka J., Jomori T. (2024). Onnamide A suppresses the severe acute respiratory syndrome-coronavirus 2 infection without inhibiting 3-chymotrypsin-like cysteine protease. J. Biochem..

[18] Jomori T., Higa N., Tyas T.A., Matsuura N., Ueda Y., Suetake A., Miyazaki S., Watanabe S., Arizono S., Hayashi Y. (2025). Onnamides and a novel analogue, Onnamide G, as potent leishmanicidal agents. Mar. Biotechnol..

[19] Miyakawa K., Matsunaga S., Yamaoka Y., Dairaku M., Fukano K., Kimura H., Chimuro T., Nishitsuji H., Watashi K., Shimotohno K. (2018). Development of a cell-based assay to identify hepatitis B virus entry inhibitors targeting the sodium taurocholate cotransporting polypeptide. Oncotarget.

[20] Volz T., Allweiss L., Ben ḾBarek M., Warlich M., Lohse A.W., Pollok J.M., Alexandrov A., Urban S., Petersen J., Lütgehetmann M. (2013). The entry inhibitor Myrcludex-B efficiently blocks intrahepatic virus spreading in humanized mice previously infected with hepatitis B virus. J. Hepatol..

[21] Fukano K., Tsukuda S., Oshima M., Suzuki R., Aizaki H., Ohki M., Park S.-Y., Muramatsu M., Wakita T., Sureau C. (2019). Troglitazone impedes the oligomerization of sodium taurocholate cotransporting polypeptide and entry of hepatitis B virus into hepatocytes. Front. Microbiol..

[22] Chang W.-W., Su I.-J., Chang W.-T., Huang W., Lei H.-Y. (2008). Suppression of p38 Mitogen-Activated Protein Kinase Inhibits Hepatitis B Virus Replication in Human Hepatoma Cell: The Antiviral Role of Nitric Oxide. J. Viral Hepat..

[23] Lai L., Li L., Cui X., Piao L., Cui Z. (2024). Chlorogenic Acid Inhibition HBV Replication by Suppressed JNK Expression. Clin. Med. Res..

[24] Li Y., He M., Gong R., Wang Z., Lu L., Peng S., Duan Z., Feng Y., Liu Y., Gao B. (2022). Forkhead O Transcription Factor 4 Restricts HBV Covalently Closed Circular DNA Transcription and HBV Replication through Genetic Downregulation of Hepatocyte Nuclear Factor 4 Alpha and Epigenetic Suppression of Covalently Closed Circular DNA via Interacting with Promyelocytic Leukemia Protein. J. Virol..

[25] Yu X., Liu C.-D., Shen S., Kim E.S., Liu Z., Zhang H., Sun N., Liu Y., Martensen P.M., Huang Y. (2025). Interferon alpha-inducible protein 27 (IFI27) inhibits hepatitis B virus (HBV) transcription through downregulating cellular transcription factor C/EBPα. J. Virol..

[26] Yao X., Xu K., Tao N., Cheng S., Chen H., Zhang D., Yang M., Tan M., Yu H., Chen P. (2024). ZNF148 inhibits HBV replication by downregulating RXRα transcription. Virol. J..

[27] Sato S., Li K., Kameyama T., Hayashi T., Ishida Y., Murakami S., Watanabe T., Iijima S., Sakurai Y., Watashi K. (2015). The RNA Sensor RIG-I Dually Functions as an Innate Sensor and Direct Antiviral Factor for Hepatitis B Virus. Immunity.

[28] Shionoya K., Park J.-H., Ekimoto T., Takeuchi J.S., Mifune J., Morita T., Ishimoto N., Umezawa H., Yamamoto K., Kobayashi C. (2024). Structural basis for hepatitis B virus restriction by a viral receptor homologue. Nat. Commun..

[29] Melegari M., Wolf S.K., Schneider R.J. (2005). Hepatitis B Virus DNA Replication Is Coordinated by Core Protein Serine Phosphorylation and HBx Expression. J. Virol..

[30] Iwamoto M., Cai D., Sugiyama M., Suzuki R., Aizaki H., Ryo A., Ohtani N., Tanaka Y., Mizokami M., Wakita T. (2017). Functional Association of Cellular Microtubules with Viral Capsid Assembly Supports Efficient Hepatitis B Virus Replication. Sci. Rep..

[31] Hirt B. (1967). Selective extraction of polyoma DNA from infected mouse cell cultures. J. Mol. Biol..

[32] Malmström S., Larsson S.B., Hannoun C., Lindh M. (2012). Hepatitis B Viral DNA Decline at Loss of HBeAg Is Mainly Explained by Reduced cccDNA Load—Down-Regulated Transcription of pgRNA Has Limited Impact. PLoS ONE.

[33] Allweiss L., Testoni B., Yu M., Lucifora J., Ko C., Qu B., Lütgehetmann M., Guo H., Urban S., Fletcher S.P. (2023). Quantification of the hepatitis B virus cccDNA: Evidence-based guidelines for monitoring the key obstacle of HBV cure. Gut.

[34] Zhang X., Zhang E., Ma Z., Pei R., Jiang M., Schlaak J.F., Roggendorf M., Lu M. (2011). Modulation of hepatitis B virus replication and hepatocyte differentiation by MicroRNA-1. Hepatology.

