# Supplementary material for: Onnamides A and B Suppress Hepatitis B Virus Transcription by Inhibiting Viral Promoter Activity
Source: Mar Drugs. 2026 Jan 1;24(1):21. doi: 10.3390/md24010021 (PMC12842730; doi:10.3390/md24010021)
Supplement: Supplementary file 1 [file marinedrugs-24-00021-s001.zip › marinedrugs-4047025-supplementary.pdf]

Supplementary Figure S1

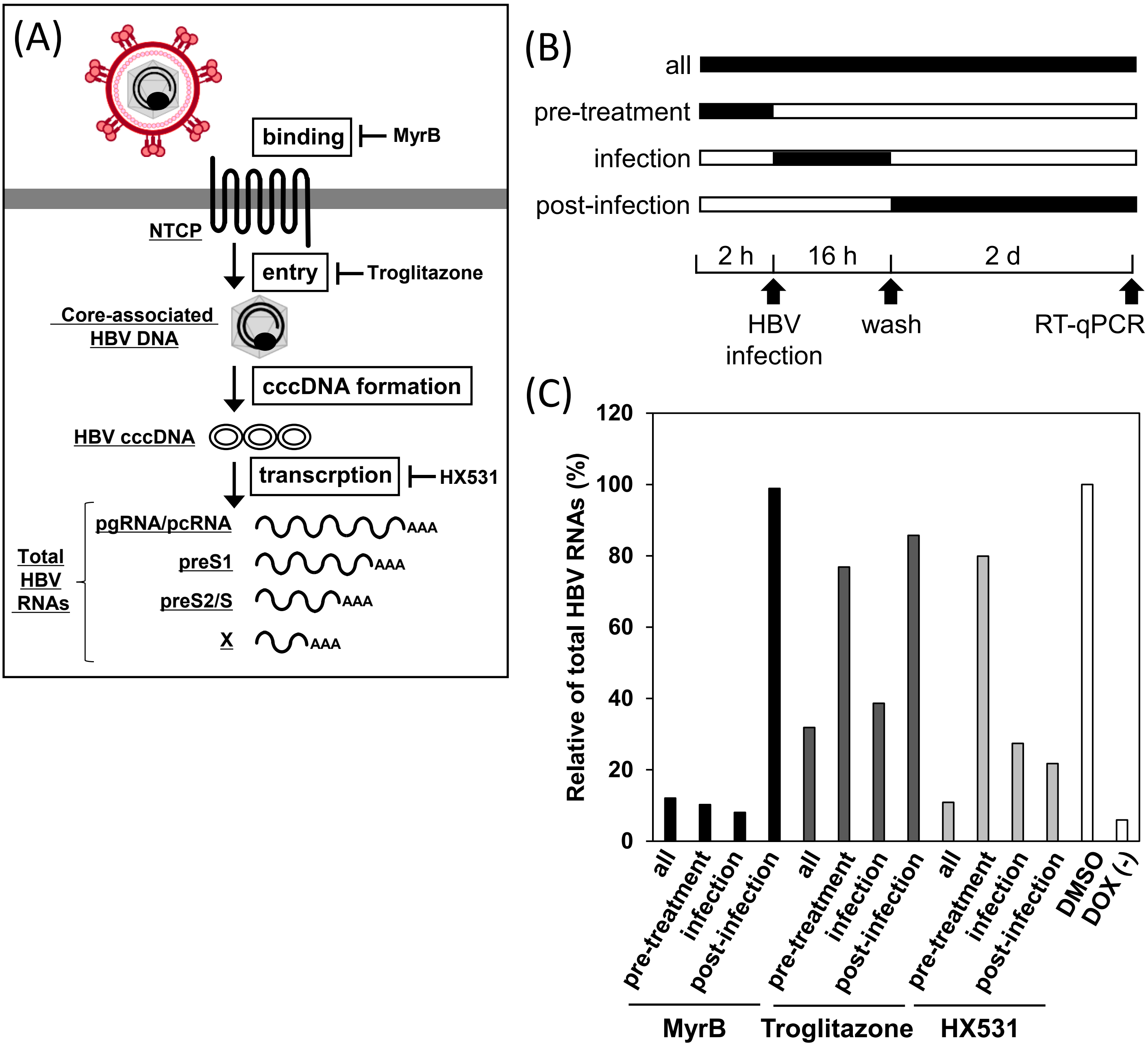

Supplementary Figure S1. Time-of-addition experiments with control compounds in iNTCP cells. (A) Schematic representation of the HBV life cycle, illustrating the steps from viral binding to transcription, with the sites of action of each compound indicated. (B) iNTCP cells were treated with the compounds before, during, or after HBV infection, as depicted in the schematic. (C) iNTCP cells were treated with MyrB (0.5 µM), troglitazone (50 µM), or HX531 (5 µM). Total HBV RNA was quantified by RT-qPCR and is presented as percentage inhibition relative to DMSO. Data represent the mean (n = 2).

Hayashi *et al*
